# An αvβ6-specific virotherapy expressing bispecific immune cell activators induces immune cell activation to mediate tumor cell death

**DOI:** 10.1016/j.omton.2025.201017

**Published:** 2025-06-25

**Authors:** Rebecca J. Bayliss, Luned M. Badder, James Davies, Andrew Robinson, Mona Pissarreck, Simon Kollnberger, Alan L. Parker

**Affiliations:** 1 Department of Cancer and Genetics, School of Medicine, Cardiff University, Cardiff CF14 4XN, UK; 2 Department of Infection and Immunity, School of Medicine, Cardiff University, Cardiff CF14 4XN, UK; 3 Systems Immunity University Research Institute, School of Medicine, Cardiff University, Cardiff CF14 4XN, UK

**Keywords:** immunotherapy, αvβ6 integrin, oncolytic, virotherapy, bispecific antibody, T cell, NK cell, solid cancers, organoid

## Abstract

Ad5_NULL_-A20 is an adenovirus type 5-based precision virotherapy engineered to selectively target αv*β*6-positive tumors. Bispecific immune cell activators (BICAs) bind both an immune cell receptor and tumor cell-associated antigen (TAA) in tandem to induce a tumor-specific immune response. Combining the selectivity and oncolytic properties of Ad5_NULL_-A20 with the potency of BICA will create a more tolerated, enduring immune cell response limited to tumor sites, reducing off-target effects and dose-limiting toxicities. We developed multiple BICA targeting T cells via CD3, natural killer (NK) cells via CD16/NKG2D receptors, and TAA epidermal growth factor receptor (EGFR) and major histocompatibility complex-related chain A (MICA). *In vitro* studies establish that Ad5_NULL_-A20 BICA in αv*β*6 tumor cells results in T cell and NK activation at tumor sites and a loss of tumor cell viability. *Ex vivo* studies validate these findings demonstrating a significant and rapid reduction in growth of patient-derived 3D tumor organoids transduced with oncolytic Ad5_NULL_-A20-BICA in the presence of T cells or NK cells. Ad5_NULL_-A20 expressing BICA can produce a potent immune response resulting in tumor eradication. This approach has significant translational potential to develop a novel cancer therapeutic for clinical success.

## Graphical abstract

**Figure:**
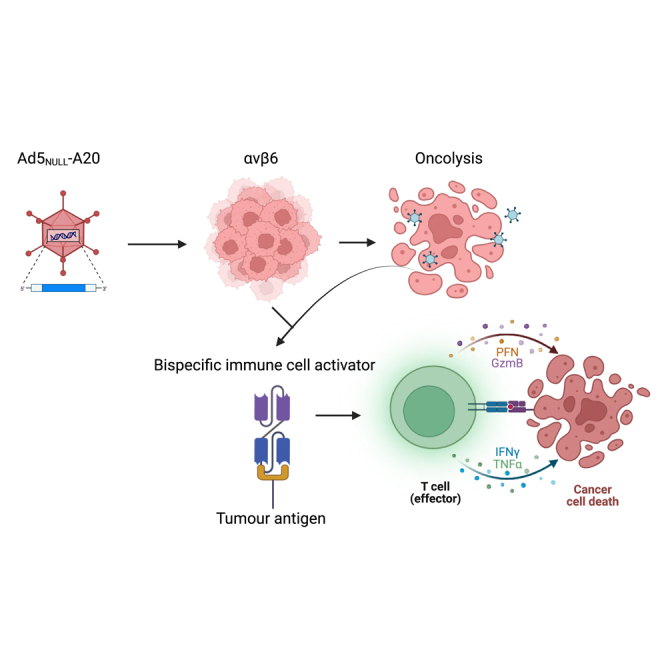

## Teaser

Virotherapies can deliver anti-cancer therapeutics directly to tumors. Bayliss and colleagues develop a precision virotherapy expressing bispecific immunotherapies from tumor cells resulting in immune cell activation and tumor cell killing. Combination cancer therapy has potential to enhance regression of solid tumors in a targeted manner improving on current approaches.

## Introduction

Solid tumors are composed of heterotypic cell masses, connective tissue, and immune cells that communicate between tight and gap junctions to enable the formation of a tumor microenvironment (TME). A vast range of immunotherapies have been designed to regulate immunostimulatory molecules within the TME to impede tumor immune escape. Often a combination of immunotherapies is used in the treatment of solid tumors to overcome tumor evasion strategies.^1^

Oncolytic viruses selectively replicate and lyse tumor cells resulting in the release of virus and tumor-specific antigens leading to immune cell infiltration at tumor sites. This in combination with the ability to carry transgenes to tumor sites makes them an attractive option for the development of cancer therapeutics.^2^ Oncolytic adenovirus type 5 (Ad5) has a good safety record and is permissive to transgene insertion; however, its efficacy as a cancer therapeutic is limited by off-target effects due to poor selectivity. To overcome such limitations, we previously developed Ad5_NULL_-A20 incorporating detargeting mutations into each of the capsid proteins, ablating native receptor binding, and reducing off-target toxicities. Ad5_NULL_-A20 was retargeted via insertion of a 20-mer A20 peptide into the fiber knob HI loop to enable selective uptake into αv*β*6-positive tumors.^3^

Bispecific immune cell activators (BICA) are traditionally composed of two single-chain variable fragments (scfv) from two individual monoclonal antibodies connected via a tandem flexible linker. BICA are designed to simultaneously bind an immune cell receptor and a tumor-associated antigen resulting in the formation of major histocompatibility complex (MHC)-independent cytolytic synapse. The formation of the synapse leads to cytotoxic release of perforin and granzyme B resulting in tumor cell lysis.^4^ BICA have shown encouraging clinical results for hematological malignancies; in 2014, the first BICA Blinatumomab (CD3×CD19) was approved to treat relapsed/refractory lymphoblastic leukemia.^5^ Treatment of solid tumors with BICA has proven to be more challenging due to limited tumor penetration and poor efficacy in the presence of the TME. The continuous systemic delivery of BICA leads to the occurrence of on-target off-tumor severe adverse events including cytokine release syndrome (CRS), neurotoxicity, and hepato- and cardiotoxicities.^6^ To date, only Tarlatamab (DLL3×CD3) has been granted Food and Drug Administration (FDA) approval for the treatment of small cell lung cancer; therefore, improvements to the delivery of BICA are paramount to significantly improve outcomes.

To enhance therapeutic potential of Ad5_NULL_-A20 in αv*β*6-positive solid epithelial tumors, we designed a range of BICA to be incorporated into the Ad5_NULL_-A20 genome allowing the release of the therapeutic at tumor sites. By harnessing the immune response generated by oncolytic infection of tumor cells, we intended to redirect and activate immune cells via the release of BICA within the local TME. This approach enables multiple tumor antigens to be targeted, generating an effective and sustained immune response at tumor sites, significantly overcoming issues associated with tumor cell heterogeneity. In addition, restricting the release of the BICA at tumor sites using a targeted virotherapy, we aim to circumvent the toxicities seen via current systemic administration, limiting damage to healthy tissue to enhance safety profile of BICA.

These studies investigate the ability of a precision virotherapy, Ad5_NULL_-A20, to transduce αv*β*6 cancer cells and produce BICA immunostimulatory molecules. Secreted BICA generated an effective immune response and reduced tumor growth in epidermal growth factor receptor (EGFR)/major histocompatibility complex-related chain A (MICA)-positive tumors *in vitro*. We confirmed this activity in pancreatic patient-derived models *ex vivo*, demonstrating a significant and rapid reduction in tumor cell growth in the presence of T cells and natural killer (NK) cells when transduced with oncolytic Ad5_NULL_-A20-BICA (OAd5_NULL_-A20-BICA).

The findings presented here support the development of OAd5_NULL_-A20-BICA as a therapeutic candidate in the treatment of more difficult to treat solids tumors. This approach enables targeting of abundant tumor-associated antigens with significant translational potential.

## Results

### Generation of Ad5_NULL_-A20-BICA with oncolytic and immunogenic properties

**Figure 1:**
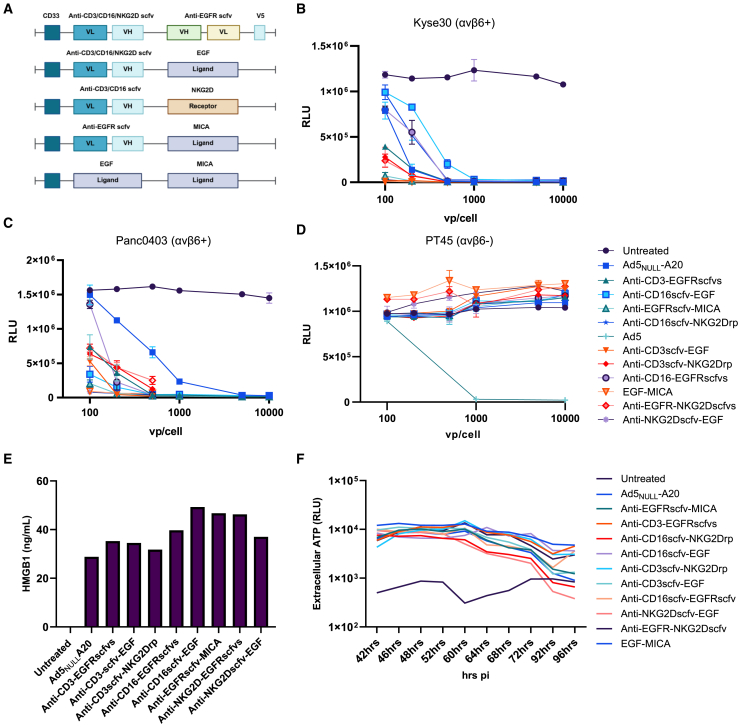
Incorporation of bispecific molecules into precision virotherapies does not affect oncolytic properties of OAd5_NULL_-A20 (A) A schematic of the bispecific molecules inserted into Ad5_NULL_-A20 virotherapy. (B–D) Comparison of oncolytic killing by OAd5_NULL_-A20-BICA in KYSE30 (αvβ6^+^) (B), Panc0403 (αvβ6^+^) (C), and PT45 (αvβ6^−^) cells (D). Mean ±SD of triplicates shown. Luminescence relative light units (RLU). (E and F) The detection of HMGB1 (E) from transduced BT20 (αvβ6^+^) cells, absolute values shown from an average of triplicates read from standard curve (F). Extracellular ATP release from UMSCC4 (αvβ6^+^) cells between 42 and 96 h, luminescence readout (RLU). Mean of triplicate values shown.

Each BICA was designed to express a human CD33 signaling peptide at the N-terminus to enable secretion from cells. Constructs containing two scfv, a variable light (VL), and variable heavy (VH) chain are displayed in a VL-VH-VH-VL format with connecting G4S N-linkers (Table S2). A V5-tagged protein at the C-terminus allows detection of scfv-scfv constructs. In the instance of a scfv-ligand and scfv-receptor format, the ligand/extracellular domain of the receptor replaces the scfv domain related to the desired target. Ligand-ligand format connects two ligands via a G4S N-linker. CD16scfv, MICA ligand, and NK group 2D (NKG2D) scfv target CD16 or NKG2D receptor (NKG2Drp) present on NK cells. CD3scfv target T cells via the CD3 receptor. Tumor antigens are in turn targeted via binding to either MICA via NKG2Drp or EGFR via EGFR scfv or epidermal growth factor (EGF) ligand (Figure 1A).

BICA were recombineered^7^ into replication-deficient and oncolytic Ad5 and Ad5_NULL_-A20 BACs and competent viruses generated (Tables S3 and S4). To determine if incorporation of the BICA transgenes detrimentally affect the virus’s oncolytic properties, three cancer cell lines, Panc0403 (αv*β*6^+^), KYSE30 (αv*β*6^+^), and PT45 (αv*β*6^−^), with variable αv*β*6 expression (Figure S1) were transduced with OAd5_NULL_-A20-BICA (Figures 1B–1D). All tested virotherapies displayed similar levels of oncolysis to OAd5_NULL_-A20 alone. Immunogenic cell death is requisite for the accumulation of damage-associated molecular patterns (DAMPs) such as ATP and HMGB1 to recruit and activate antigen-presenting cells (APC). One major advantage of oncolytic viruses in the treatment of cancer is the ability to induce immunogenic cell death at tumor sites. To establish if OAd5_NULL_-A20-BICA induce immunogenic cell death in αv*β*6^+^ cells, HMGB1 release was quantified after 24 h transduction (Figure 1E). An increase in HMGB1 between 30 and 50 ng/mL was evident in all Ad5_NULL_-A20-BICA transduced cells equivalent or greater than the Ad5_NULL_-A20 control. Extracellular ATP release was evident for all OAd5_NULL_-A20-BICA peaking at 60–64 h post-infection and gradually declining at 96 h, coinciding with cell death (Figure 1F). These data indicate that incorporation of bispecific transgenes does not significantly impact oncolytic nor immunogenic properties of OAd5_NULL_-A20, ensuring optimal performance of the precision virotherapy at tumor sites.

### BICA activation of immune cells is dependent on EGFR and MICA tumor antigen binding

**Figure 2:**
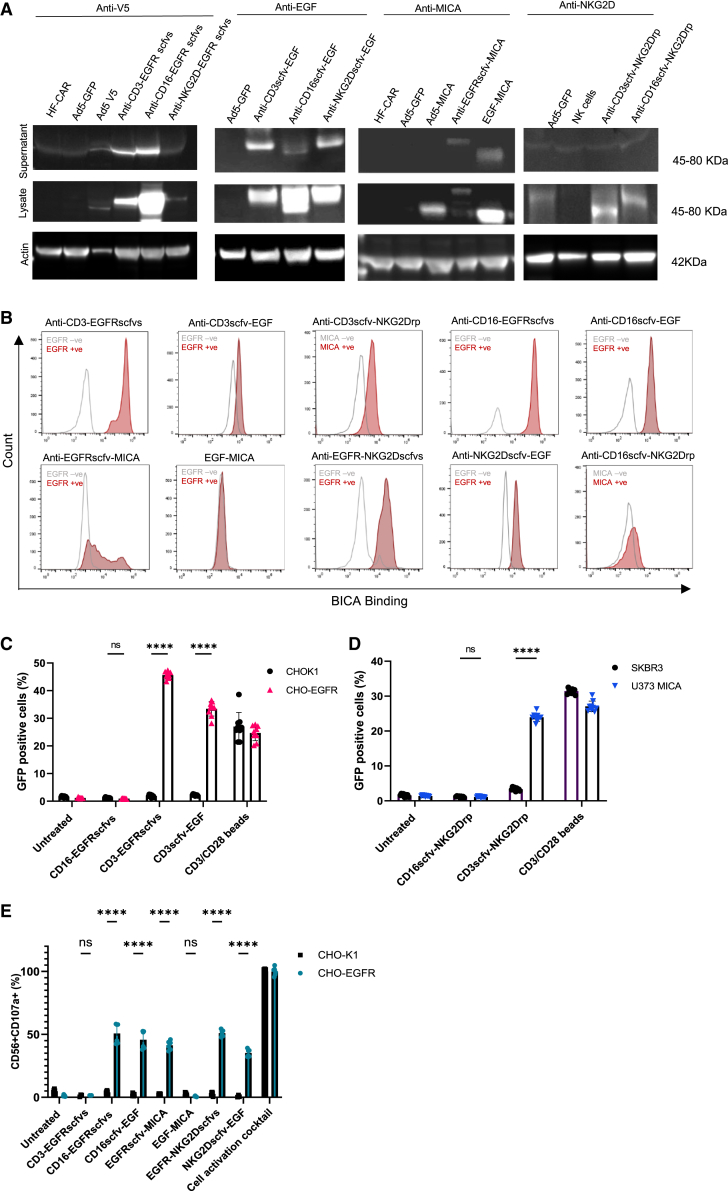
Secreted BICA bind to EGFR/MICA-positive cells and activate immune cells (A) Western blot analysis of the expression and secretion of BICA constructs. (B) Binding of BICA to target tumor antigens. CHOK1 (EGFR−), A341 (EGFR+) or CHO-EGFR (EGFR+), SKBR3 (MICA−), and U373-MICA (MICA +) were incubated with BICA supernatants prior to addition of corresponding labeled recombinant protein or antibody; fluorescent signal was detected via flow cytometry. (C and D) CD3-BICA induce activation of Jurkat NF-κB GFP reporter cells. (C) Percentage of GFP +ve Jurkat NF-κB cells co-cultured with CD3-BICA supernatants in CHOK1 (EGFR−) and CHO-EGFR (EGFR+) cells. CD3/CD28 antibody beads are used as a positive control. An off-target BICA (CD16-EGFRscfvs) was used as a negative control. Mean ±SD shown (*n* = 3). ∗∗∗∗*p* < 0.0001 and ns, not significant. (D) Percentage of GFP +ve Jurkat NF-κB cells co-cultured with CD3-BICA supernatants in SKBR3 (MICA−) and U373-MICA (MICA+) cells. CD3/CD28 antibody beads were used as a positive control. An off-target bispecific supernatant (CD16scfv-NKG2Drp) was used as a negative control. Mean ±SD of individual values shown (*n* = 3). ∗∗∗∗*p* < 0.0001 and ns, not significant. (E) NK-targeting BICA induce activation of NK cells. Percentage of CD107a-positive NK cells co-cultured in the presence of CD16-BICA supernatants in CHOK1 (EGFR−) and CHO-EGFR (EGFR+) cells. Cell activation cocktail was used as a positive control. An off-target BICA (CD3-EGFRscfvs) was used as a negative control. Mean ±SD shown of triplicates (*n* = 2). ∗∗∗∗*p* < 0.0001 and ns, not significant.

Expression and secretion of BICA from transduced cells is paramount to their function to activate immune cells at tumor sites. Supernatants containing secreted BICA, and cell lysates were processed for western blotting. Variable expression of all BICA molecules was evident within cell lysates. The majority of BICA were secreted at detectable levels, except for CD16/CD3scfv-NKG2Drp (Figures 2A and S2).

Binding to respective tumor cell-associated antigen (TAA) was assessed by incubating supernatants containing BICA on EGFR/MICA-positive or -negative cell lines (Figures 2B, S3, and S4). Binding to EGFR or MICA was evident for eight out of ten BICA tested when compared to TAA-negative cells. CD16scfv-NKG2Drp showed no binding to MICA, whereas EGF-MICA did not show any binding to EGFR.

The biological activity and specificity of CD3-BICA were initially evaluated *in vitro* using Jurkat nuclear factor κB (NF-κB) green fluorescent protein (GFP) reporter cells incubated with the various CD3-BICA supernatants (Figures 2C and 2D) in the presence or absence of EGFR or MICA (Figure S3). NF-κB-driven transcription of GFP resulting from binding to CD3 was significantly increased in the presence of CD3-EGFRscfvs, CD3scfv-EGF, and CD3scfv-NKG2Drp, dependent on the presence of TAA. No GFP was evident in the absence of TAA or in the presence of an “off-target” BICA. In the case of CD3-EGFRscfvs GFP induction was higher than the positive control CD3/CD28 beads (Figure 2C). Using a similar approach, NK cells targeting BICA supernatants were co-cultured with NK-derived cells in the presence or absence of EGFR (Figure 2E). A substantial increase in CD56^+^CD107a^+^ cells, indicative of NK cell activation, was evident in five of six BICA tested. The exception being EGF-MICA where no increase in CD107a was detected.

These data confirm that T cell and NK cell BICA specifically and efficiently bind to tumor antigens in an EGFR/MICA-dependent manner and engage with target immune cells to induce an effective immune response. Seven of the BICA evaluated were selected for further analysis: CD3-EGFRscfvs, CD3scfv-EGF, CD16-EGFRscfvs, CD16scfv-EGF, EGFRscfv-MICA, EGFR-NKG2Dscfvs, and NKG2Dscfv-EGF. Although secretion of CD3scfv-NKG2Drp was not evident (Figure 2A), the BICA performed well in other assays leading to the decision to take it forward for further testing. EGF-MICA was detected via western blot using the MICA antibody; however, it failed to show evidence of binding when targeted to EGFR, suggesting the EGF ligand was not binding as expected. Similarly, CD16scfv-NKG2Drp failed to show any binding or functionality, likely due to poor protein expression and/or processing. Therefore, these were both removed from further analysis.

### Ad5_NULL_-A20 CD3-BICA induce T cell activation and immune-mediated tumor death

**Figure 3:**
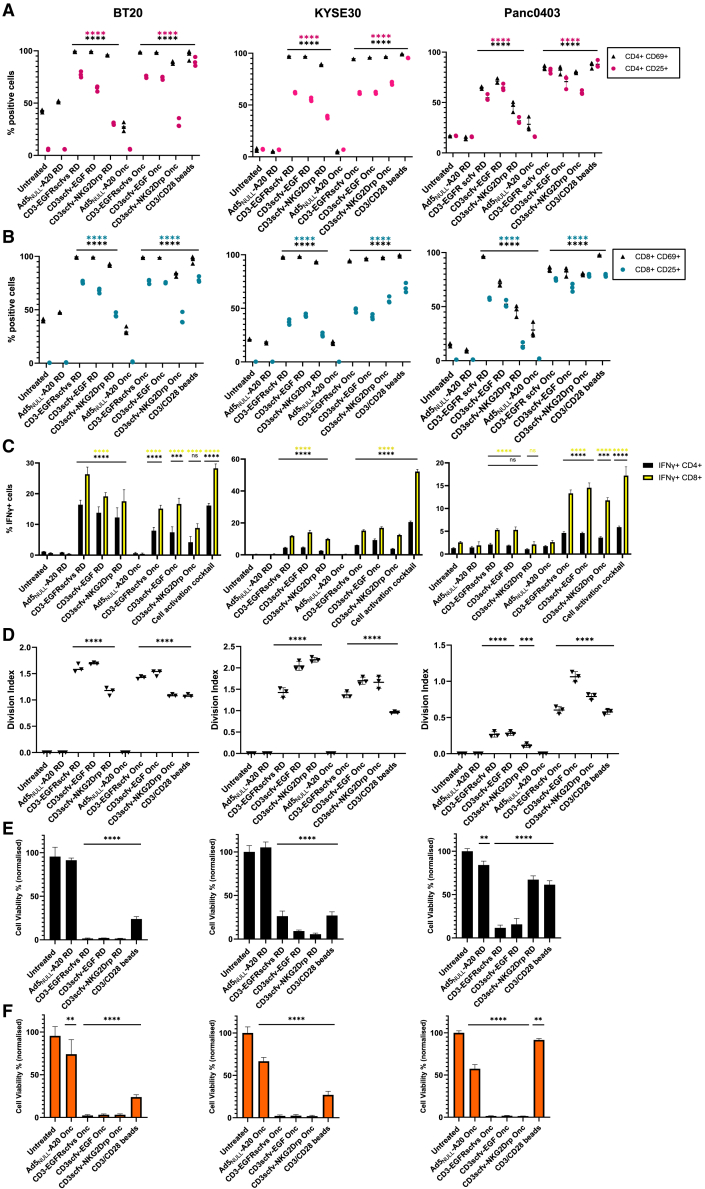
Ad5_NULL_-A20 expressing CD3-BICA induces T cell activation and immune-mediated killing of cancer cells (A and B) CD4^+^ and CD8^+^ T cell activation by CD3-BICA in BT20, KYSE30 and Panc0403. CD3^+^ T cells were co-cultured with Ad5_NULL_-A20 CD3-BICA-transduced cancer cells and percentage of CD25- and CD69-positive CD4^+^ (A) and CD8^+^ (B) T cell subsets measured by flow cytometry. (C) Intracellular IFN-γ. Percentage of IFN-γ-positive T cells 48 h post co-culture. (D) T cell proliferation. Measured over for 5 days by CD3^+^ T cells pre-loaded with proliferation dye and analyzed by flow cytometry. Cell division index of CD3^+^ T cells was calculated using FlowJo software. All experiments (A–D) were performed in two independent donors: one representative donor shown. Mean ±SD of triplicates shown. (E and F) Immune cell-mediated killing of cancer cell lines expressing BICA. Cell viability of transduced cells with Ad5_NULL_-A20 CD3-BICA co-cultured with CD3^+^ T cells. Ad5_NULL_-A20 replication-deficient 1–5 days (E) and oncolytic (F) 1–2 days post-co-culture. Viability normalized to untreated. Experiments performed in two independent donors: one representative donor shown. Mean ±SD of triplicates repeats shown. Statistical significance was assessed versus untreated cells by two-way ANOVA followed by Dunnett’s post hoc analysis for (A–C) and one-way ANOVA followed by Dunnett’s post hoc test for (D–F) (ns *p* > 0.05, ∗*p* < 0.05, ∗∗*p* < 0.01, ∗∗∗*p* < 0.001, and ∗∗∗∗*p* < 0.0001). RD, replication-deficient; Onc, oncolytic.

Ad5_NULL_-A20 selectively targets αvβ6-positive solid epithelial tumors; therefore, three cancer cell lines representing a range of epithelial solid tumor types were selected: BT20 (triple-negative breast cancer), KYSE30 (esophageal squamous cell carcinoma), and Panc0403 (pancreatic adenocarcinoma). Each cell line overexpresses αvβ6, required for Ad5_NULL_-A20 transduction, and EGFR and MICA (including other NKG2D ligands) required for BICA activation (Figure S1 and Table S1), therefore possess the required characteristics to demonstrate T cell activation and tumor cell lysis by CD3-BICA. CD25 and CD69 early activation markers were used to determine activation of the T cells via CD3 in CD3-BICA transduced cells (Figures 3A and 3B). A clear increase in both CD69 and CD25 was evident in CD4^+^ and CD8^+^ T cell populations transduced with Ad5_NULL_-A20-CD3-BICA compared to controls. CD3-EGFRscfvs and CD3scfv-EGF saw the greatest increase (CD69 98%–99% and CD25 60%–80%) with activation, and in many cases, equivalent to the positive control. In the instance of CD3scfv-NKG2Drp, activation was lower, particularly in the replication-deficient virotherapies; this is in line with the fact that CD3-EGFRscfvs and CD3scfv-EGF are more highly expressed and secreted than CD3scfv-NKG2Drp (Figure 2A) in cells. A similar pattern of activation was evident in both KYSE30 and Panc0403 cells. However, Panc0403 transduced with replication-deficient Ad5_NULL_-A20-BICA RD had lower CD69-positive T cells compared to BT20 and KYSE30.

Intracellular interferon gamma (IFN-γ) increased in the presence of Ad5_NULL_-A20-CD3-BICA transduced cells compared to controls, with CD3-EGFRscfvs and CD3scfv-EGF performing better than CD3scfv-NKG2Drp. Intracellular IFN-γ was more elevated in CD8^+^ T cells than CD4^+^ T cells across the board, with BT20 cells seeing a greater overall increase in IFN-γ-positive cells than KYSE30 or Panc0403 (Figure 3C). T cell proliferation over five days also saw an increase in the CD3^+^ T cell division index compared to controls (Figure 3D). Panc0403 transduced with replication-deficient Ad5_NULL_-A20 CD3-BICA had notably reduced proliferation compared to the oncolytic.

A dramatic loss of cell viability (100%–75%) in both replication-deficient (Figure 3E) and oncolytic (Figure 3F) cells transduced with Ad5_NULL_-A20 CD3-BICA was evident between 1 and 5 days co-culture, except for Panc0403 CD3scfv-NKG2Drp, which only reduced cell viability by 33% compared to untreated cells. The reduced activity of Ad5_NULL_-A20 CD3-BICA RD in Panc0403 cells is attributed to low secretion levels of the BICA in this cell line, which is overcome by the use of an oncolytic alternative.

Altogether, these data provide evidence that Ad5_NULL_-A20 CD3-BICA virotherapies can, when transduced into αvβ6-positive tumor cells, release CD3-BICA at the tumor site resulting in activation of T cells and downstream proliferation amounting to a potent immune response at tumor sites to induce immune-mediated lysis of tumor cells.

### Ad5_NULL_-A20 CD16-BICA induce NK activation and immune-mediated tumor death

**Figure 4:**
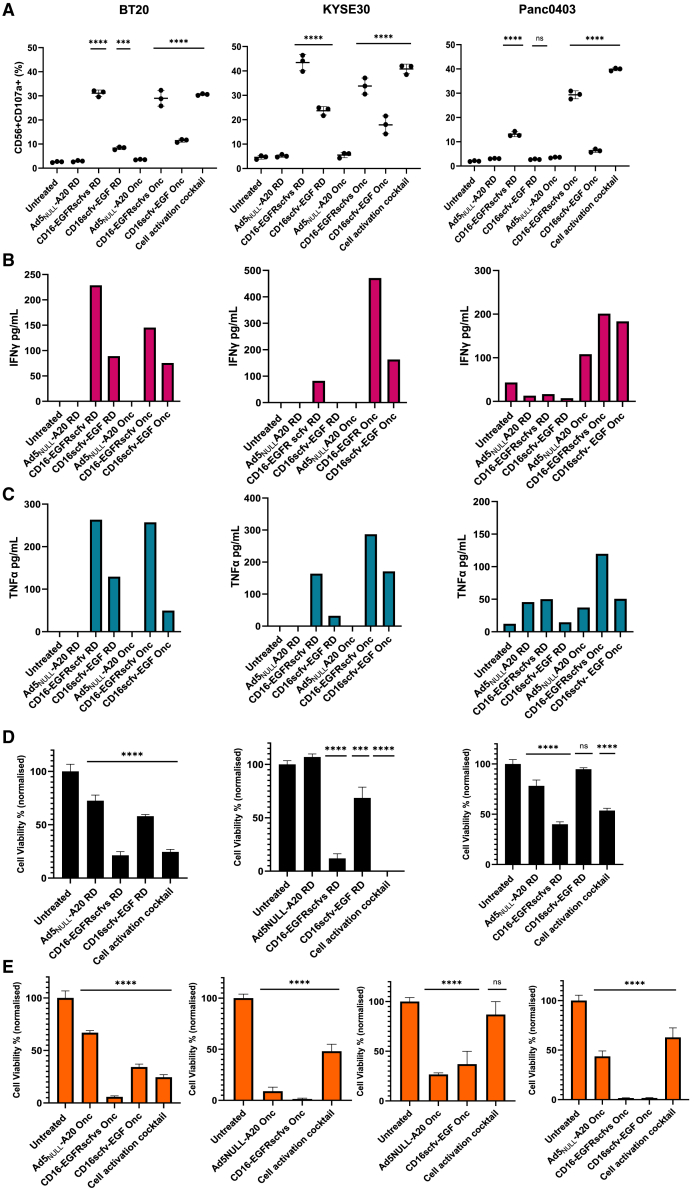
Ad5_NULL_-A20 expressing CD16-BICA induces NK cytotoxicity and immune-mediated killing of cancer cells (A) NK cytotoxicity. Percentage of CD56^+^ CD107^+^ PBMC co-cultured with Ad5_NULL_-A20 CD16-BICA transduced BT20, KYSE30, and Panc0403 analysis of the NK cell subset by flow cytometry. Experiments performed in two independent donors: one representative donor shown. Mean ±SD of triplicates shown. (B and C) IFN-γ (B) and TNF-α (C) production in supernatants of cancer cells transduced with Ad5_NULL_-A20 CD16-BICA and co-cultured with PBMC. (D and E) NK cell-mediated killing of cancer cell lines transduced with Ad5_NULL_-A20 CD16-BICA. Cell viability of cancer cells expressing CD16-BICA and co-cultured with NK cells. Ad5_NULL_-A20 replication-deficient, 1–5 days post-co-culture (D) and oncolytic 1–2 days post-co-culture, performed as two independent experiments (E). Viability normalized to untreated. Experiments performed in two independent donors: one representative donor shown. Mean ±SD of triplicates repeats shown. Statistical significance was assessed versus untreated cells one-way ANOVA followed by Dunnett’s post hoc test for (A, D, and E) (ns *p* > 0.05, ∗*p* < 0.05, ∗∗*p* < 0.01, ∗∗∗*p* < 0.001, and ∗∗∗∗*p* < 0.0001). RD, replication-deficient; Onc, oncolytic.

CD107a or lysosome-associated membrane protein-1 (LAMP-1) is a marker for degranulation of NK cells to determine cytotoxic activity; therefore, αvβ6-positive cells were transduced with Ad5_NULL_-A20-expressing NK-BICA and co-cultured with peripheral blood mononuclear cells (PBMCs). A significant increase in NK degranulation was evident in NK cell co-cultured with tumor cells transduced with Ad5_NULL_-A20 CD16-EGFRscfvs (approximately 30%–40%). CD16scfv-EGF producing cells saw a more modest increase of between 7% and 22% (Figure 4A). Replication-deficient Ad5_NULL_-A20 CD16-BICA showed reduced CD107^+^ levels in Panc0403 in comparison to the oncolytic as seen previously in the CD3-BICA.

Remaining constructs (EGFRscfv-MICA, EGFR-NKG2Dscfvs, and NKG2Dscfv-EGF) saw no increase in CD107a degranulation over background levels (not shown). Further testing of supernatants containing BICA (Figures S5A and S5B) showed an increase of CD107a^+^ positive cells (25%–50%) when normalized to negative control BICA (Figure S5B), with comparable results to initial screening (Figure 2E), suggesting the BICA can bind and activate NK cells. We next evaluated this in the context of Ad5_NULL_-A20-BICA transduction co-cultured with purified NK cells. A modest increase in CD107a^+^ cells was seen in replication-deficient transduced cells (10%–15%) compared to untreated cells, suggesting low secretion levels of the BICA from transduced cells. In contrast, cells transduced with OAd5_NULL_-A20-BICA saw an increase (40%–45%) (Figure S5C) equivalent to previous levels (Figure S5A); however, OAd5_NULL_-A20 devoid of any transgene also induced an increase of around 30%. Taken together, these data suggest that EGFRscfv-MICA, EGFR-NKG2Dscfvs, and NKG2Dscfv-EGF as standalone BICA can function by inducing NK cell cytotoxicity; however, OAd5_NULL_-A20 is also able to trigger NK degranulation. Although the combination of the BICA does marginally increase the response, it is difficult to discern the benefits of the addition of the bispecific to OAd5_NULL_-A20 alone.

Tumor necrosis factor alpha (TNF-α) and IFN-γ play important roles in surveillance of tumor growth, therefore TNF-α and IFN-γ production from NK cells co-cultured with Ad5_NULL_-A20 CD16-BICA transduced cells was measured by ELISA. IFN-γ and TNF-α levels increased in transduced BT20 cells with both replication-deficient and OAd5_NULL_-A20 CD16-BICA compared to controls. CD16-EGFRscfv saw a greater increase in cytokine production (15–28 pg/mL) than CD16scfv-EGF which produced around 1- to 3-fold less IFN-γ (Figure 4B) and TNF-α (Figure 4C). KYSE30 and Panc0403 also saw a similar increase in IFN-γ and TNF-α production (50–480 pg/mL) in cells transduced with OAd5_NULL_-A20 CD16-BICA; however, replication-deficient Ad5_NULL_-A20 CD16-BICA cytokine production was noticeably lower (0–100 pg/mL) for both IFN-γ and TNF-α.

Cell viability was determined between 1 and 5 days co-culture with purified NK cells in the presence of Ad5_NULL_-A20 CD16-BICA (Figures 4D and 4E). A loss of cell viability over 95% was evident in cells transduced with OAd5_NULL_-A20 CD16-EGFRscfvs, greater than Ad5_NULL_-A20 alone, suggesting NK cell-mediated killing of the cancer cells. Replication-deficient Ad5_NULL_-A20 CD16-EGFRscfvs reduced cell viability between 60% and 90% dependent on cell line. Despite moderate increases in CD107a, TNF-α, and IFN-γ for cells transduced with OAd5_NULL_-A20 CD16scfv-EGF, a 65% decrease in BT20 and KYSE30 viability was observed, increasing to 100% in Panc0403. Replication-deficient Ad5_NULL_-A20 CD16scfv-EGF reduced BT20 and KYSE30 viability by 30%–40%; however, >10% killing was seen in Panc0403.

Taken together, these data indicate that Ad5_NULL_-A20 CD16-EGFRscfvs is a potent activator of NK cells inducing cytotoxicity and release of TNF-α and IFN-γ resulting in NK cell-induced tumor cell death. Ad5_NULL_-A20 CD16scfv-EGF, although functional, exhibits a reduced level of cytotoxicity and tumor cell death in comparison to CD16-EGFRscfvs due to low expression levels in cells (Figure 2A). Despite this, CD16scfv-EGF in the oncolytic background still has potential to be a viable BICA moving forward; therefore, OAd5_NULL_-A20 CD16-EGFRscfvs and CD16scfv-EGF were taken forward for further testing.

### OAd5_NULL_-A20 CD3-BICA induce T cell-mediated tumor cell death in an *ex vivo* human pancreatic organoid model

3D patient-derived tumor organoids retain many phenotypic and genetic properties of parental tumors, making them a valuable tool for the screening of cancer therapeutics. Characterization of pancreatic ductal adenocarcinoma (PDAC) organoids shows expressions of the surface receptors αv*β*6 and EGFR (Figure S6A), both required for internalization of Ad5_NULL_-A20 and bispecific tumor antigen targeting, respectively, making them a suitable model for demonstrating the potency of OAd5_NULL_-A20-BICA *ex vivo*. Negligible MICA was found on the surface of the organoids tested; however, solubilized MICA was present in the supernatants at high levels in PDM38 and PDM36 (Figure S6C).

**Figure 5:**
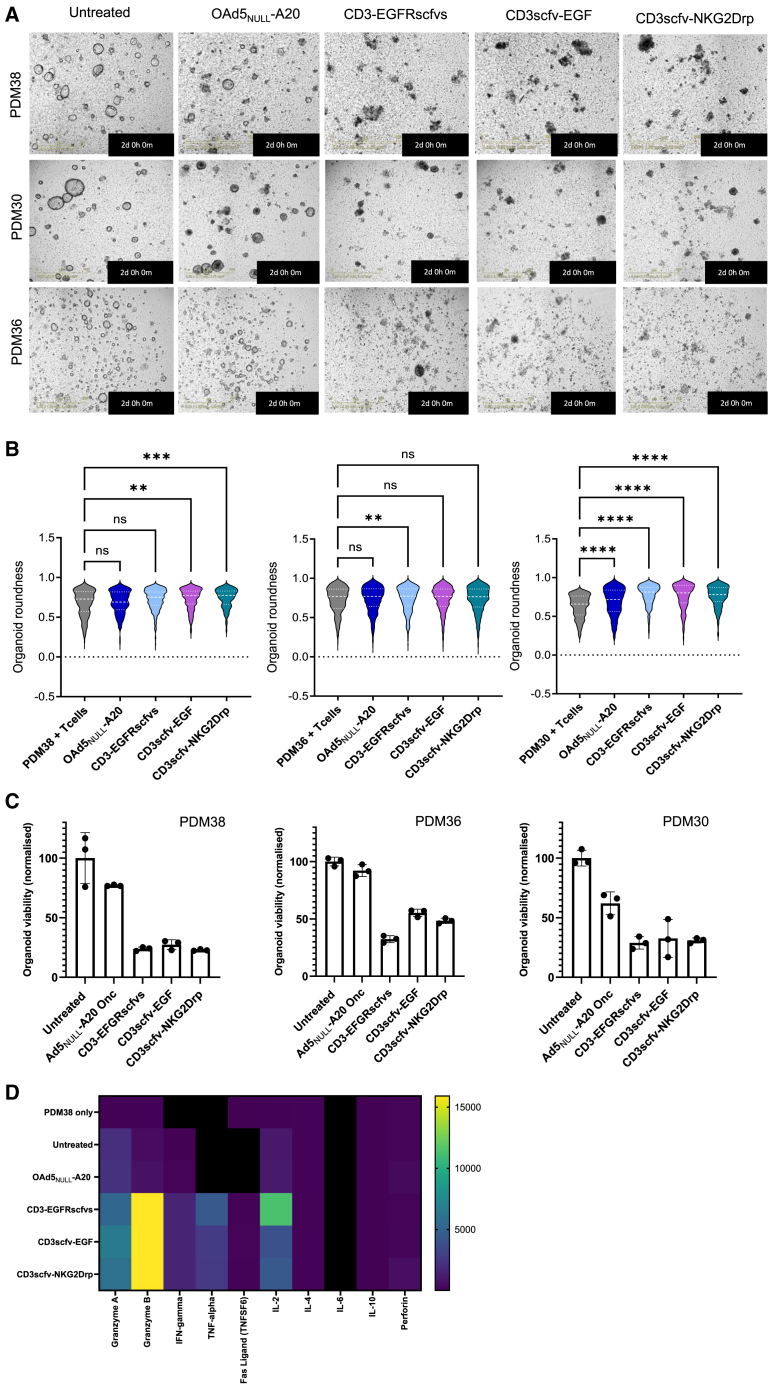
Ad5_NULL_-A20 expressing CD3-BICA promote T cell-mediated killing of patient-derived pancreatic organoids (A) Representative IncuCyte images of pancreatic organoids transduced with OAd5_NULL_-A20 or OAd5_NULL_-A20 CD3-EGFRscfvs, CD3scfv-EGF, and CD3scfv-NKG2Drp in co-culture with CD3^+^ T cells 2 days post-infection. Performed in 2 independent donors, one representative donor shown for each sample. Scale bars, 100 μm. (B) IncuCyte analysis of organoid integrity using a measure of “roundness” in (A). 0 = round/maximum integrity. One-way ANOVA was used to compare across groups. ∗∗*p* < 0.01, ∗∗∗*p* < 0.001, and ∗∗∗∗*p* < 0.0001; ns, not significant. (C) Measure of cell viability at 72 h post-co-culture. Performed in 2 independent donors, one representative donor shown for each sample. Mean ± SD of triplicate repeat samples shown. One-way ANOVA followed by Dunnett’s post hoc test was used to assess (C) (ns *p* > 0.05, ∗*p* < 0.05, ∗∗*p* < 0.01, ∗∗∗*p* < 0.001, and ∗∗∗∗*p* < 0.0001). (D) Luminex xMAP multiplex cytokine analysis of PDM38 co-cultures, one representative donor shown. Displayed as a heatmap indicating increases in cytokine production (pg/mL).

PDAC organoids from three donors (Table S5) were transduced with OAd5_NULL_-A20-CD3-BICA before co-culture with CD3^+^ T cells. The anti-tumor activity of OAd5_NULL_-A20 CD3-BICA was evaluated against untreated and OAd5_NULL_-A20 transduced cells co-cultured with T cells. T cell killing of organoids transduced with OAd5_NULL_-A20 CD3-BICA can be seen as early as 18 h, peaking at 48 h post-co-culture across the three co-cultures (Figure 5A). Untreated cells and OAd5_NULL_-A20 remain viable at this time point and continue to grow in the presence of the T cells. Analysis of organoid images show a decrease in organoid roundness, synonymous with reduced organoid integrity, in organoids transduced with OAd5_NULL_-A20 CD3-EGFRscfvs, CD3scfv-EGF, and CD3scfv-NKG2Drp compared to controls (Figure 5B). In addition, labeling of T cells shows clear infiltration of the organoid cultures compared to controls (Figure S7). Cell viability was measured at 72 h post-infection confirming the enhanced cell death seen in OAd5_NULL_-A20 CD3-BICA transduced organoids compared to Ad5_NULL_-A20 alone (Figure 5C). Luminex cytokine analysis of co-culture supernatants indicate heightened production of granzyme B, TNF-α, IFN-γ, interleukin (IL)-2 and low levels of Fas ligand and perforin (Figure 5D) suggestive of T cell cytotoxicity. In contrast, IL-6 was not detectable within these samples and IL-10 was only detected in one sample (PDM38) at very low levels, which was not consistent across samples.

### OAd5_NULL_-A20 CD16-BICA induce NK cell-mediated tumor cell death in an *ex vivo* human pancreatic organoid model

**Figure 6:**
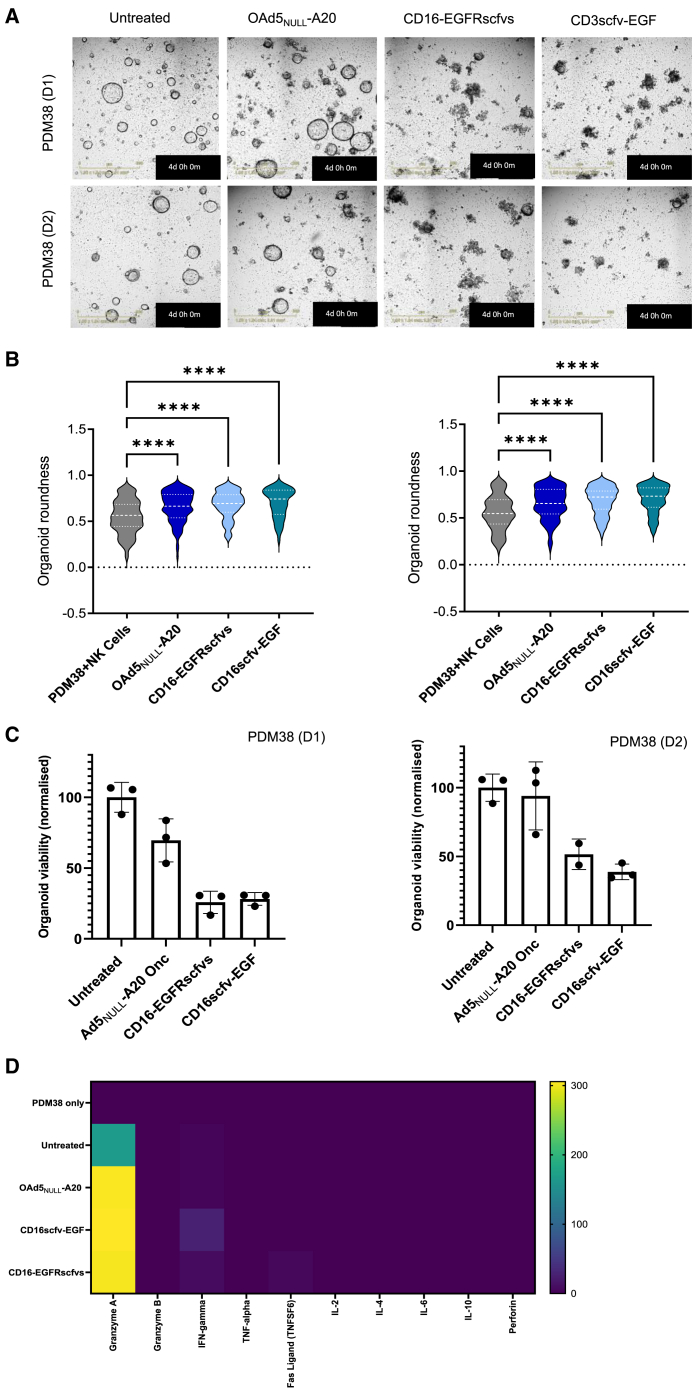
Ad5_NULL_-A20 expressing CD16-BICA promote NK cell-mediated killing of patient derived pancreatic organoids (A) Representative IncuCyte images of pancreatic organoid (PDM38) transduced with OAd5_NULL_-A20 or OAd5_NULL_-A20 CD16-EGFRscfvs, CD16scfv-EGF in co-culture with NK cells (E:T 1:8) at 72 h post-infection. Two independent NK cell donors shown. (B) IncuCyte analysis of organoid integrity using a measure of “roundness” in (A). 0 = round/maximum integrity. One-way ANOVA was used to compare across groups. ∗∗∗∗*p* < 0.0001. (C) Measure of cell viability at 96 h post-co-culture. Shown in 2 independent NK cell donors. Mean ± SD of triplicate repeat samples shown. One-way ANOVA followed by Dunnett’s post hoc test was used to assess (C) (ns *p* > 0.05, ∗*p* < 0.05, ∗∗*p* < 0.01, ∗∗∗*p* < 0.001, and ∗∗∗∗*p* < 0.0001). (D) Luminex xMAP multiplex cytokine analysis of PDM38 co-cultures, one representative donor shown. Displayed as a heatmap indicating increases in cytokine production (pg/mL).

NK cell’s ability to reduce tumor organoid growth in the presence of CD16-BICA was assessed over 4 days using the IncuCyte. OAd5_NULL_-A20 CD16-EGFRscfvs, and CD16scfv-EGF induced comparable NK cell-mediated killing of organoids with a distinct increase in cell death evident around 72 h post-co-culture when compared to untreated and OAd5_NULL_-A20. As expected, OAd5_NULL_-A20 also induced NK cell killing of transduced organoids around 72 h, with organoids appearing smaller, with a distinct loss of structure and integrity; however, the rate of tumor cell regression was noticeably slower in the absence of CD16-BICA (Figure 6A). In line with these observations, a significant decrease in organoid roundness was evident in organoids transduced with OAd5_NULL_-A20 CD16-BICA compared to controls (Figure 6B). In addition, organoids viability was compromised compared to controls (Figure 6C) in conjunction with an increase in granzyme A and IFN-γ (Figure 6D). In the context of an *ex vivo* organoid model, OAd5_NULL_-A20 CD16-EGFRscfvs and CD16scfv-EGF transduction results in NK cell-mediated death suggesting that a targeted virotherapy in conjunction with a CD16-EGFR BICA can effectively inhibit tumor cell growth.

Overall, these data provide evidence that OAd5_NULL_-A20 CD3/CD16-BICA actively increase T cell and NK cell anti-tumor activity, resulting in enhanced cell death in αv*β*6/EGFR/MICA-positive PDAC models, suggesting their therapeutic benefit in a wide range of solid tumors with the same phenotypic profile.

## Discussion

The development of bispecific antibodies has revolutionized cancer immunotherapy, outperforming the clinical efficacy of monoclonal antibodies.^8^ The non-IgG-like format results in a shorter half-life (2 h) with better tissue-penetration, lower rate of resistance, less immunogenicity, and increased immune specificity.^9^^,^^10^^,^^11^ However, these advances are limited for the treatment of solid tumors due to the necessity to administer via continuous intravenous infusion, resulting in off-target toxicities, potentially leading to CRS in patients.^12^

The combination of BICA with oncolytic virotherapies enables the generation of BICA *in situ* offering many therapeutic advantages including selective replication in tumor cells reducing damage to healthy tissue and promoting tumor infiltrating lymphocytes (TILS) and remodeling and evasion of the immunosuppressive TME. Introduction of a dual modality of tumor cell killing by oncolysis or BICA encourages penetration of oncolytic viruses (OV) into solid tumors, targeting heterogeneous tumors, and reducing systemic toxicities by restricting BICA production to transduced cancer cells while overcoming limitations of the BICA short half-life.^13^ Ad5_NULL_-A20 offers additional benefits by selectively targeting αvβ6 integrin-expressing solid tumors with minimal off-target effects that restrict current Ad5-based therapies.^3^ In addition, Ad5_NULL_-A20 has the potential to be delivered intravenously, widening its therapeutic potential for the treatment of metastasis, which is limited by intra-tumoral delivery.^14^

Antibody resistance to existing bispecific antibodies targeting a single TAA is currently a significant limitation leading to target cells losing sensitivity in turn resulting in tumor recurrence. OAd5_NULL_-A20-BICA aims to overcome this limitation by targeting two separate tumor antigens αvβ6 and EGFR simultaneously. Nonetheless, immune escape is still a possibility and exploration of armed OVs in combination with tri-specific T cell engagers targeting two TAA concurrently^15^ or complementary therapies should be explored. Previous research shows the DNA damage response induced by radiotherapy and chemotherapy increases surface expression of NKG2D ligands, enhancing activity of a herpes virus simplex OV expressing a NKG2Drp bispecific.^16^ Such synergistic effects could be exploited by Ad5_NULL_-A20 CD3-NKG2Drp BICA in combination with either radiotherapy or chemotherapy.

The activation of a large number of T cells by BICA represents a challenge to reach optimal therapeutic potential while reducing toxic side effects. Although our data confirm a robust activation of T cells exposed to CD3-BICA and complete tumor cell killing for all cell lines tested within 24 h (oncolytic) or 5 days (replication-deficient) (Figure 3), it should be noted that *in vitro* experiments use homogeneous cell lines with 100% transduction efficiency, which is unlikely to be achieved in heterogeneous tumors. Organoid cultures also saw rapid T cell-mediated killing as early as two days (Figure 5) in all CD3-BICA tested, though more representative of patient samples, we predict a much more measured response *in vivo*. By delivering the CD3-BICA in Ad5_NULL_-A20 directly to the tumor, it is highly unlikely enough BICA would be produced locally to induce the toxicities currently seen via continuous systemic delivery. Encouragingly, previously developed Ad5-based vectors containing T cell targeting BICA^17^ have produced promising results undergoing preclinical evaluation: ICOVIR-15K armed with an anti-EGFR × CD3 BiTE^18^ and engineered oncolytic group B adenovirus enadenotucirev (EnAd) armed with an anti-EpCAM × CD3 BiTE,^19^ paving the way for development of more adenovirus-based combination therapies. Like many OVs, Ad5_NULL_-A20 can broaden its therapeutic potential through the design of BICA targeting alternative TAA such as EPCAM and HER2, for applications in cancers that lack EGFR or MICA. As Ad5_NULL_-A20 is inherently more tumor selective at the level of cell recognition, we predict increased potency with negligible off-target effects *in vivo*.

NK cells have generated increasing interest as a solid cancer therapeutic target in recent years due their attractive anti-tumor properties; they demonstrate lower toxicity and higher safety than T cell BICA, and therefore lower CRS and off-target toxicity.^20^ For example, an Anti-CD16 × CD33 bispecific antibody for the treatment of myelodysplastic syndrome (MDS) has been reported to eradicate CD33^+^ MDS cells and targeted CD33^+^ myeloid-derived suppressor cells resulting in reduced immunosuppression in the TME and enhanced antitumor efficacy.^21^ Here, we demonstrate anti-CD16-EGFR BICA produces an NK-mediated cytotoxic effect when expressed in various cancer cell lines *in vitro*, although the resulting NK-mediated tumor cell killing is not as potent as seen with the CD3-BICA in replication-deficient transduced cells; we see complete killing with the addition of the oncolytic (Figure 4). *Ex vivo* data showed NK cell-mediated killing of PDAC organoids over 4 days (Figure 6), twice as much time as CD3-BICA; this more measured response holds promise for a longer lasting more robust immune response at tumor sites suggesting Ad5_NULL_-A20 CD16-BICA has great potential to be an effective, well-tolerated therapeutic going forward.

Organoid models show great promise in predicting patient response to treatment.^22^ Using these models in combination with immune cells adds another level of complexity, demonstrating Ad5_NULL_-A20-BICA ability to work in synergy with immune cells creating a potent anti-tumor response in a relevant, complex heterogeneous system.

Ad5_NULL_-A20 CD3-NKG2Drp BICA targets MICA on tumor cells. We show Ad5_NULL_-A20 CD3-NKG2Drp BICA increases T cells activation *in vitro* albeit at lower levels than CD3-EGFR/EGF (Figure 3), which correlates with lower MICA expression on the cell lines tested in this study (Figure S1). Interestingly, we also saw consistent tumor cell killing of PDAC organoids *ex vivo* (Figure 5) despite very low levels of MICA on the organoid surface (Figure S5A); however, detectable levels of soluble MICA in organoid supernatants were present (Figure S5C), suggesting MICA is being cleaved from the tumor surface as previously reported in pancreatic cancer,^23^ which did not appear to impact the BICA functionality. In addition to MICA, NKG2D has several MHC-I-like ligand-binding partners including MICB and ULBP1–6, as with MICA, these ligands are typically expressed at low levels on the surface of healthy cells but can be upregulated during oncogenic transformation.^2018^ Receptor staining of organoid cultures with these additional ligands (Figure S5B) showed expressions of ULBP2 (>25%) and MICA (>10%). PDM36, PDM38, and PDM30 had very low MICA (>2%); however, ULBP1, 2, and 4 were detected at low levels (>10%) on both organoids, suggesting CD3-NKG2Drp BICA may be binding to additional ligands in addition to MICA resulting in T cell activation. Additionally, we cannot rule out that transduction with Ad5_NULL_-A20 itself may be driving increased expression of NKG2D ligands through the DNA damage response.^24^ Overall, we hypothesize that CD3scfv-NKG2Drp could be used to target multiple NKG2D ligands on transformed cells in addition to MICA adding an additional benefit to its therapeutic potential.

Despite the heterogenic nature of the organoid samples, the combination of αvβ6 targeting oncolytic virus and EGFR/MICA targeting BICA resulted in >50% regression in organoid viability across the organoid donors tested (Figures 5 and 6). Tumor regression correlated with an increase in cytokine production evident in both T cell and NK (Figures 5D and 6D) co-cultures, suggesting that the delivery of the BICA via Ad5_NULL_-A20 to tumors enhances T cell and NK cytotoxicity. Overall, these experiments point toward Ad5_NULL_-A20-BICA inducing a pro-immunogenic TME with potential to kill tumors devoid of target TAAs αvβ6, EGFR, and/or MICA.

Overcoming the hostile TME and stroma surrounding tumors remains a challenge to improve the dissemination of OVs to target tumor cells. Encouragingly, research is making promising progress in this area by adopting multiple approaches including delivery of OV via the use of mesenchymal stromal cells,^25^ targeting the stroma itself with the use of armed OVs,^26^ and using tumor sensitizers such as histone deacetylase inhibitors^27^^,^^28^ to enhance OV delivery to target sites. As the delivery of immunotherapies via OVs improves and moves to the clinic, a more refined approach to the delivering of viral transgenes should be explored, such as the use of tumor-specific enhancers/promoters to further control gene expression and improve patient safety.^27^

In summary, we demonstrate that the local delivery of T cell or NK cell targeting BICA in combination with Ad5_NULL_-A20 produces an effective anti-tumor response in αv*β*6-positive tumors resulting in T cell and NK cell cytotoxicity and tumor regression. This provides an exciting and potentially highly effective approach to systemically target cancer immunotherapies while overcoming the limitations associated with the current systemic delivery of BICA. As OAd5_NULL_-A20 heads to clinical trials, it will be of great interest to see how combination therapies using this method of delivery develop in the future and how they will best fit into current treatment regimes. Ad5_NULL_-A20-BICA shows promise as a potent viro-immunotherapy that could be readily transferred to the clinic in the near future.

## Material and methods

### Cell culture

T-Rex-293, HEK293-β6, HF-CAR, SKBR3, A431, UMSCC4, and PT45 were maintained in DMEM. U373-MICA YFP, KYSE30, Panc0403, and Jurkat NF-κB GFP reporter cells were maintained in RPMI 1640 (Sigma). CHO-K1 were maintained in DMEM-F-12 media and BT20 in MEM, alpha modification (Sigma). Basal media was supplemented with 10% fetal bovine serum (FBS), heat inactivated, 1% L-glutamine (200 mM stock), 2% penicillin and streptomycin (Sigma). CHO expressing EGFR (CHO-EGFR) were generated in-house using Flp-in system (Invitrogen) and maintained with the addition of 500 μg/mL hygromycin (Invitrogen) and 293-β6 with the addition of 1.25 μg/mL puromycin (Sigma). See Table S1 for additional cell line characteristics.

### Immune cell isolation

Apheresis cones obtained from Welsh Blood Service (Talbot Green, South Wales) and used under local University ethics approval. Apheresis cones were processed by layering whole blood onto Ficoll-Paque Plus (Cyvita) following manufacturer’s instructions. Immune cell isolation was carried out by magnetic activating cell sorting (MACS) using MACS isolated CD3^+^ T cells (Pan T cell isolation kit, Miltenyi Biotec) or NK cells (NK isolation kit, Miltenyi Biotec) and cultured in supplemented RPMI plus IL-2 or IL-15, respectively.

### Generation of viral vectors

Constructs were designed in Snapgene software (v.6.2.1) (Table S1) and plasmids generated by Thermo Fisher Scientific. Ad5_NULL_-A20 bacterial artificial chromosome (BAC) was generated previously in-house^3^ (Table S2). BICA were incorporated into the Ad5_NULL_-A20 E1/E1A region using AdZ recombineering as previously described.^3^^,^^7^ Viral titers were determined by MicroBCA (1 μg protein = 4 × 10^9^ virus particles [vp]) (Thermo Fisher Scientific), plaque assay and Nanosight technology (NS300, Malvern).

### Cell receptor staining

A total of 100,000 cells were stained with primary antibodies human anti-αvβ6 (Millipore), anti-MICA (OriGene), anti-EGFR-PE (BioLegend), and appropriate IgG Isotype controls (IgG1, Abcam and IgG2a-PE, BioLegend) for 1 h on ice. Where relevant, secondary antibody Alexa 647 labeled goat anti-mouse F(ab’)^2^ (Life Technologies) was applied to cells (1 h, on ice), prior to fixation in 4% paraformaldehyde (PFA) (Sigma) and analyzed by flow cytometry (Accuri C6, BD).

### Cell viability assays

Twenty-five thousand cells were transduced with a dilution (100–5,000 vp/cell) of oncolytic Ad5_NULL_-A20 with or without BICA transgenes and incubated for 5 days. To assess immune-mediated killing of cancer cells, 25,000 cells/well were transduced with Ad5_NULL_-A20 bispecific 2,000 vp/cell (replication-deficient) or 500 vp/cell (oncolytic). Virus was removed and replaced with complete media after 3 h. After 48 h, purified CD3^+^ or NK cells were added to the transduced cells and incubated for a further 24–120 h. Cell viability was determined by CellTiter-Glo luminescent cell viability assay (Promega).

### Immunogenicity assays

Cells were seeded at 20,000 cells/well and transduced with 5,000 vp/cell OAd5_NULL_-A20-BICA. Extracellular adenosine triphosphate (ATP) release was detected by addition of RealTime-Glo extracellular ATP release assay reagent (Promega) and readings taken every 6 h. HMGB1 release was calculated using Lumit High-mobility group box 1 (HMGB1) immunoassay (Promega) at 24 h post-transduction.

### Western blotting

HF-CAR cells were transduced with replication-deficient Ad5-BICA (MOI, 10) for 72 h. Supernatants were collected and cell lysate generated by lysing cell pellets in RIPA buffer (Thermo Fisher Scientific). Samples were run on 4%–12% Bis-Tris NuPAGE gel (Thermo Fisher Scientific) and transferred to Nitrocellulose membrane (GE Healthcare) and blocked before addition of either anti-V5 tag (Bio-Rad), anti-MICA (OriGene), anti-EGF (R&D Systems), or anti-NKG2D (Thermo Fisher Scientific) antibodies. Primary antibodies were detected with an anti-mouse-IgG linked horseradish peroxidase (HRP) secondary antibody (Merck). Membranes containing lysate samples were re-probed with anti-Actin (Bio-Rad) followed by Rabbit anti-IgG-HRP (Bio-Rad). Protein densitometry was performed using ImageJ (v.1.45). Lysate displayed as relative abundance to actin loading control.

### EGFR/MICA binding assay

CHOK1 (EGFR−ve) and A431 (EGFR+ve) cells were incubated with CD3-EGFRscfvs, CD16-EGFRscfvs, and EGFR-NKG2Dscfvs supernatants containing secreted BICA molecules for 1 h on ice followed by addition of an anti-V5 tag antibody and an anti-mouse secondary labeled with Alexa Fluor 647. Alternatively, EGFRscfv-MICA and EGF-MICA were incubated on CHOK1/CHO-EGFR cells and detected via an anti-MICA antibody (OriGene). In the instance of CD3scfv-EGF, CD16scfv-EGF, and NKG2Dscfv-EGF, 0.5 μg of His-tagged recombinant CD3, CD16, or NKG2D (ACROBiosystems) replaced primary antibodies followed by Anti-His Alexa Fluor 647 (BioLegend). MICA +/− cells (SKBR3 and U373-MICA, respectively) were incubated with CD3scfv-NKG2Drpand CD16scfv-NKG2Drp supernatants before addition of corresponding recombinant protein. Labeled cells were detected via flow cytometry.

### Jurkat NF-κB GFP reporter assay

Twenty-five thousand tumor cells were co-cultured with Jurkat NF-κB GFP reporter cells (System Biosciences) with an effector-to-target ratio of 1:5 (E:T 1:5) in presence supernatants containing secreted CD3-BICA from transduced cells, for 24 h. Dynabeads CD3/CD28 (Gibco) were used as a positive control (1:1 ratio). Percentage of GFP-positive cells were determined by flow cytometry.

### NK CD107a assay

Twenty-five thousand CHOK1/CHO-EGFR were co-cultured with NK cell lines,^29^ (E:T 1:2) in the presence of supernatants containing secreted NK-BICA for 6 h with CD107a-FITC (BioLegend), Golgi-Plug (BD), and GolgiStop (BD). Cell activation cocktail (BioLegend) was added to positive control wells at 1:50. NK cells were stained with LIVE/DEAD Aqua stain (Invitrogen) and CD56-BV605 (BioLegend), and fixed in 4% PFA. For PBMC co-culture assays, a total of 50,000 cells in a 24-well plate were transduced with Ad5_NULL_-A20 BICA (replication-deficient at 2,000 vp/cell or oncolytic at 500 vp/cell). After 72 h, cells were co-cultured with PBMC (E:T 1:10) or isolated NK cells (E:T 1:2) and CD107a as mentioned previously, and PBMC stained with LIVE/DEAD fixable Aqua, CD14-BV510, CD19-BV510, CD3-BV711, and CD56-BV506 before analysis by flow cytometry.

### T cell assays

A total of 25,000 cells were transduced with Ad5_NULL_-A20 CD3-BICA at 2,000 vp/cell (replication-deficient) or 500 vp/cell (oncolytic) for 48 h. CD3^+^ T cells were added at E:T 1:5. Dynabeads Human T-Activator CD3/CD28 (Life Technologies) were used as a positive control at a 1:1 ratio unless otherwise stated. T cells were stained with LIVE/DEAD fixable Aqua stain, anti-CD3-PECy7, anti-CD4-FITC, anti-CD8-PEFire700, anti-CD25BV711, and anti-CD69-Alexa-Fluor-647 (AF647) (BioLegend) in activation assays. Intracellular IFN-γ assays co-cultured T cells for 6 h in the presence of Brefeldin A (GolgiPlug, BD). Cell Activation Cocktail (BioLegend) was added to control wells at 1:50. T cells were stained with LIVE/DEAD Aqua, anti-CD3-PECy7, anti-CD4-FITC, and anti-CD8-PEFire700 followed by fixation and permeabilization using the BD Cytofix/Cytoperm kit (Invitrogen) before addition of anti-IFN-γ-APC (BioLegend). To monitor T cell proliferation, CD3^+^ T cells were pre-incubated with Cell Trace Far red cell proliferation kit (Invitrogen) prior to co-culture for 5 days. All samples were analyzed by flow cytometry.

### Flow cytometry analysis

All raw data obtained from Attune NxT and Accuri C6 was analyzed using FlowJo v.10 software. Cells were gated on single cells using LIVE/DEAD followed by gating on lineage markers were applicable. Fluorescence minus one (FMO) or isotype controls were used as reference for setting the gates. T cell proliferation was analyzed using FlowJo cell proliferation function and model adapted to best fit of the data. The percentage of cells positive for specific markers or division indexes were plotted in GraphPad Prism software, version 8.1.2.

### TNF-α and IFN-γ ELISA

Cells (25,000 cells/well) were transduced with Ad5_NULL_-A20-BICA before the addition of purified NK cells (E:T 1:2). Supernatants were collected at 48 h post-co-culture. Human TNF-α and IFN-γ levels were analyzed using DuoSet ELISA development system (R&D Systems) following manufacturer’s instructions.

### Patient-derived organoids

Models and data were derived from the Human Cancer Models Initiative (HCMI) https://ocg.cancer.gov/programs/HCMI; dbGaP accession no. phs001486. PDAC organoids (Table S5) were cultured as recommended in ATCC formulation 3 (full media). Receptor staining was carried out on single cell suspensions, and EGF was removed from the media 3 days prior to staining. For co-culture assays, organoids were harvested 2–3 days after seeding (∼100 μM diameter). Organoids were transduced in suspension with 500–1,000 vp/cell OAd5_NULL_-A20-BICA and incubated for 30 min at 37°. Isolated CD3^+^ T cells (E:T 1:10) or (NK cells E:T 1:8) were co-cultured with organoids in 30% Matrigel (Corning). Matrigel was allowed to polymerize, and then overlaid with full media without EGF. When indicated, T cells were labeled with CFSE cell proliferation dye according to manufacturer’s instructions (Life Technologies). Plates were incubated in IncuCyteS3 (Sartorius). Image acquisition was set for every 3 h for a duration of between 3 and 5 days. Brightfield images and image analysis was performed either using the IncuCyte organoid module or standard settings in the presence of fluorescent labeled cells (Sartorius). Luminex xMAP cytokine custom analysis of co-culture supernatants collected at the end of each experiment was conducted by Indoor Biotechnologies, Inc., Cardiff, UK.

### Statistics

Data were analyzed using GraphPad Prism (GraphPad Software). Mean and standard deviation (SD) shown unless otherwise stated. Two-way ANOVA multiple comparison with Dunnett’s test to compare treatments was used unless otherwise stated. ∗∗*p* < 0.01, ∗∗∗*p* < 0.001, and ∗∗∗∗*p* < 0.0001; ns, not significant.

## Data availability

Data are available within the manuscript and upon reasonable request from the corresponding author.

## Acknowledgments

The authors are grateful to Professor Richard Stanton for providing access to IncuCyte, HF-CAR cells, and general recombineering advice; Dr. Ceri Fielding for providing MICA/B, ULBP antibodies, U373-MICA YFP cells, and MICA advice; Professor Arwyn Jones for providing A431 and SKBR3 cells (all at Cardiff University, Cardiff, UK); and Indoor Biotechnologies for conducting Luminex analysis of samples. Graphical abstract and Figure 1A were created in BioRender.com (figure codes: p6qzru4 and h74ic82, respectively). Anonymous healthy donor blood was acquired from Welsh Blood Service and used under local University ethics approval. Funding was provided by Cancer Research UK Biotherapeutic Programme award to A.L.P. (C52915/A29104) and Cancer Research UK Experimental Cancer Medicine Centre award to Cardiff University (C7838/A25173).

## Author contributions

R.J.B., conceptualization, methodology, visualization, investigation, formal analysis, writing – original draft, and editing. L.M.B., investigation, methodology, formal analysis, and editing. S.K., methodology, investigation, and editing. J.D., conceptualization, methodology, and investigation. A.R., investigation. M.P., investigation. A.L.P., conceptualization, visualization, resources, funding acquisition, writing – original draft and editing, and study supervision.

## Declaration of interests

A.L.P. is CSO of Trocept Therapeutics, part of Accession Therapeutics Ltd.

## Supplemental information

Document S1. Figures S1–S7 and Tables S1–S5

Document S2. Article plus supplemental information
