# Supplementary material for: An αvβ6-specific virotherapy expressing bispecific immune cell activators induces immune cell activation to mediate tumor cell death
Source: Mol Ther Oncol. 2025 Jun 25;33(3):201017. doi: 10.1016/j.omton.2025.201017 (PMC12307681; doi:10.1016/j.omton.2025.201017)
Supplement: Document S1. Figures S1–S7 and Tables S1–S5 [file mmc1.pdf]

OMTON, Volume *33*

Supplemental information

An $\alpha v\beta 6$-specific virotherapy expressing bispecific immune cell activators induces immune cell activation to mediate tumor cell death

Rebecca J. Bayliss, Luned M. Badder, James Davies, Andrew Robinson, Mona Pissarreck, Simon Kollnberger, and Alan L. Parker

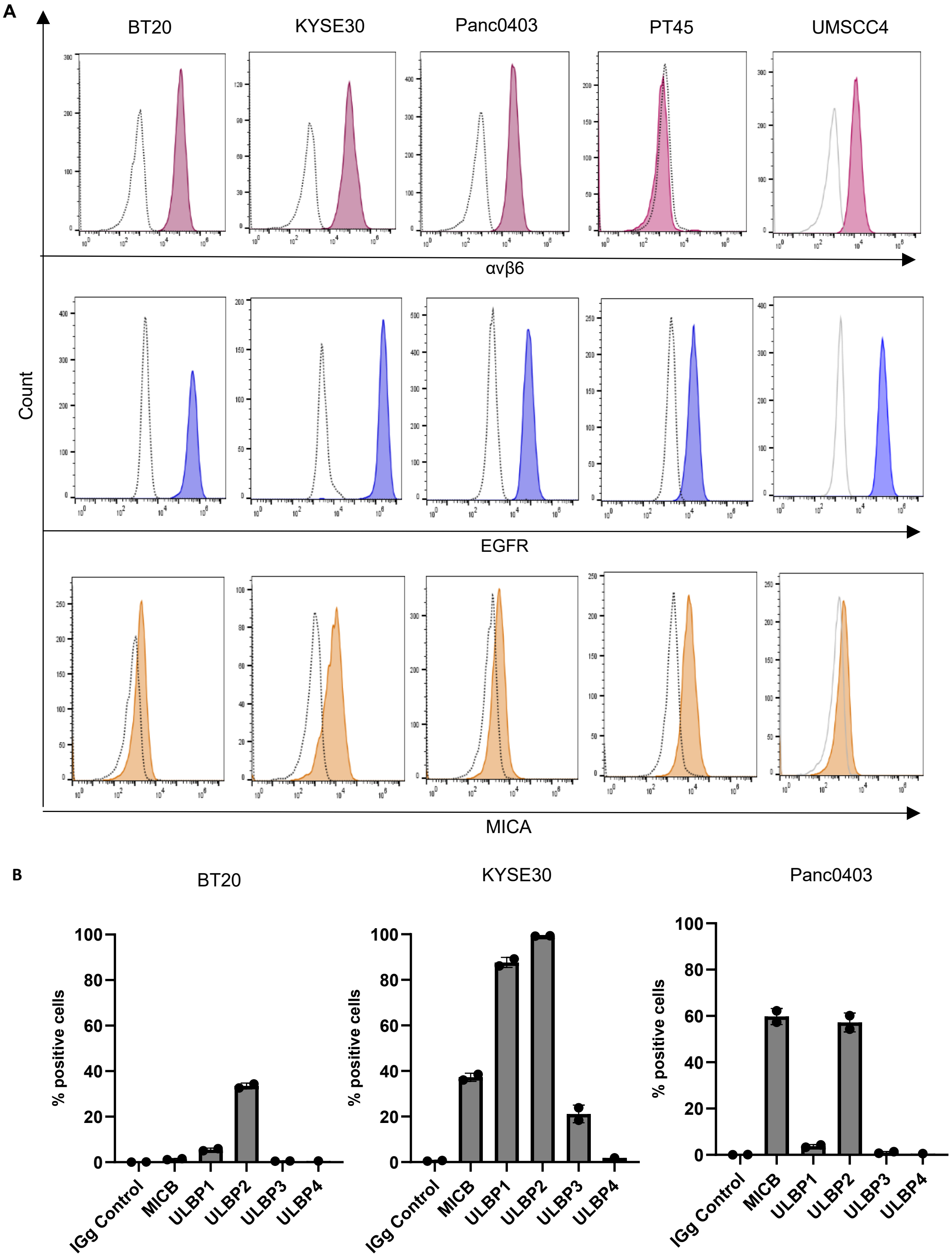

Figure S1. αvβ6, EGFR, MICA phenotyping of Cancer cell lines in co-culture experiments. (**A**) BT20, KYSE30, Panc0403, PT45 and UMSCC4 were stained for surface receptors αvβ6, EGFR, and MICA and analysed by flow cytometry. Dotted black line: IgG isotype control, pink: αvβ6, purple: EGFR, orange: MICA. **(B)** BT20, KYSE30 and Panc0403 were stained for NKG2D ligands MICB, ULBP1-4 and analysed by flow cytometry.

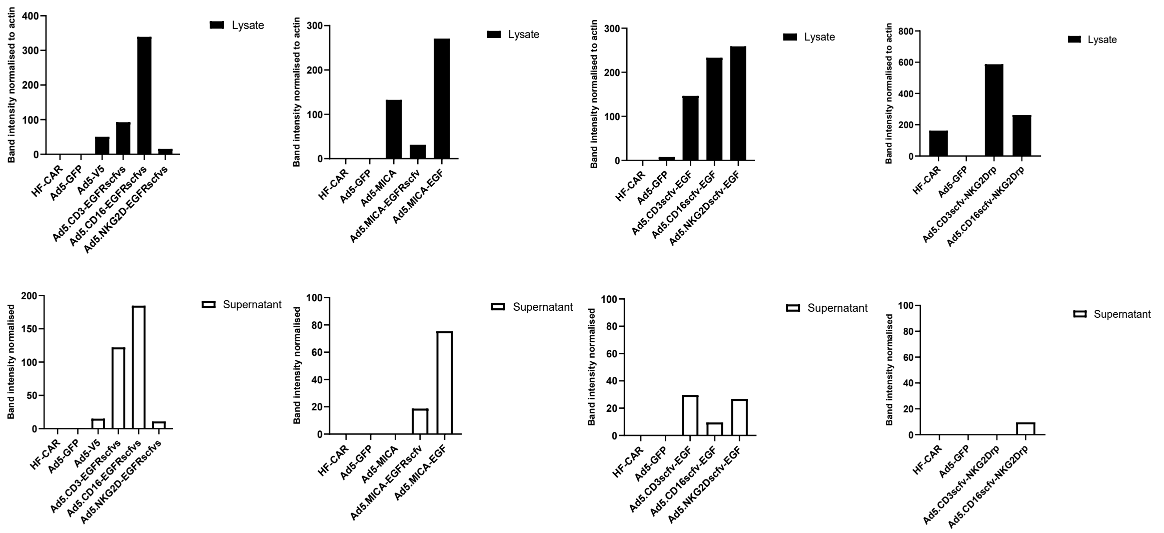

Figure S2. Western blot densitometry. Band density was quantified for cell lysates by measurement of the relative abundance of protein compared to Actin control. Data was further normalized to cell only (HF-CAR) control. Supernatant banding density was measured and normalized to cell supernatant (HF-CAR).

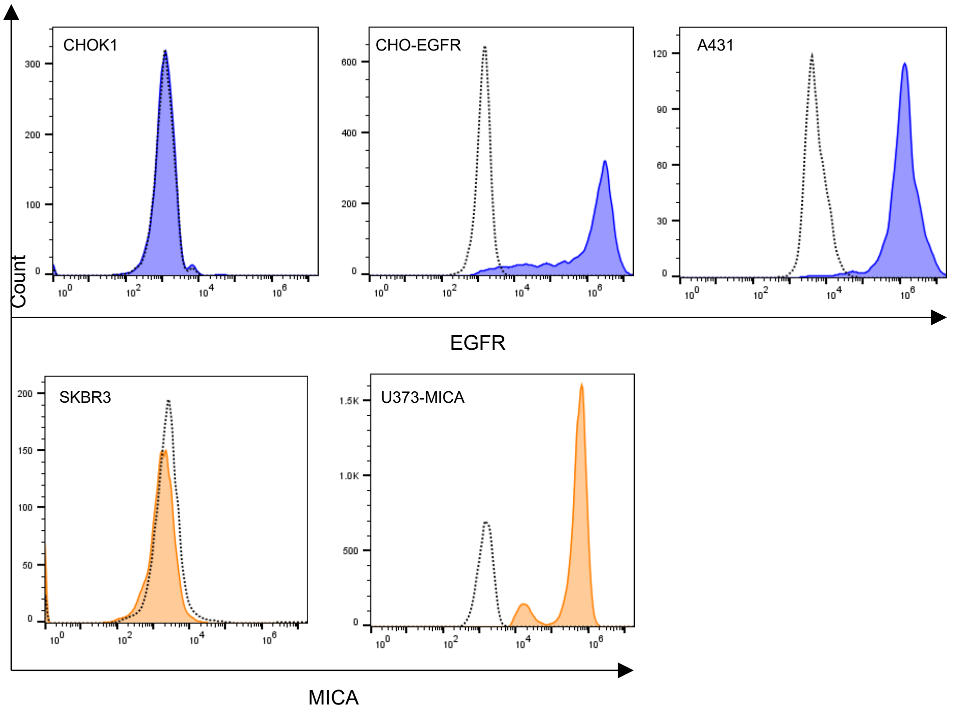

Figure S3. EGFR and MICA phenotyping of cell lines used for BICA validation. CHOK1, CHO-EGFR, A431 were stained for EGFR, SKBR3 and U373-MICA stained for MICA

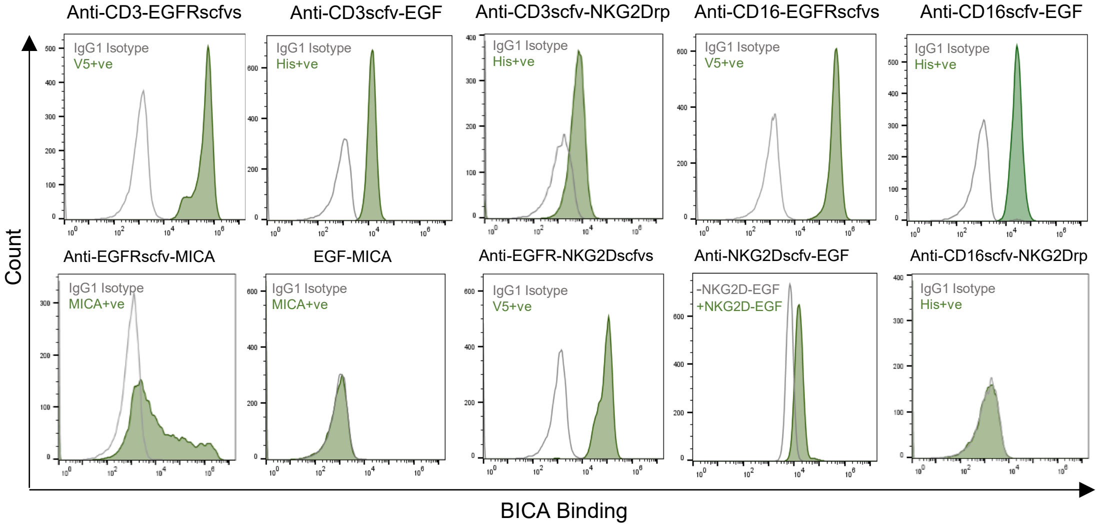

Figure S4. Binding of BICA to tumour antigens isotype controls. Binding of BICA supernatants to EGFR (A431/CHO-EGFR) and MICA (U373-MICA) positive (+ve) cells compared to IgG1 isotype control antibody.

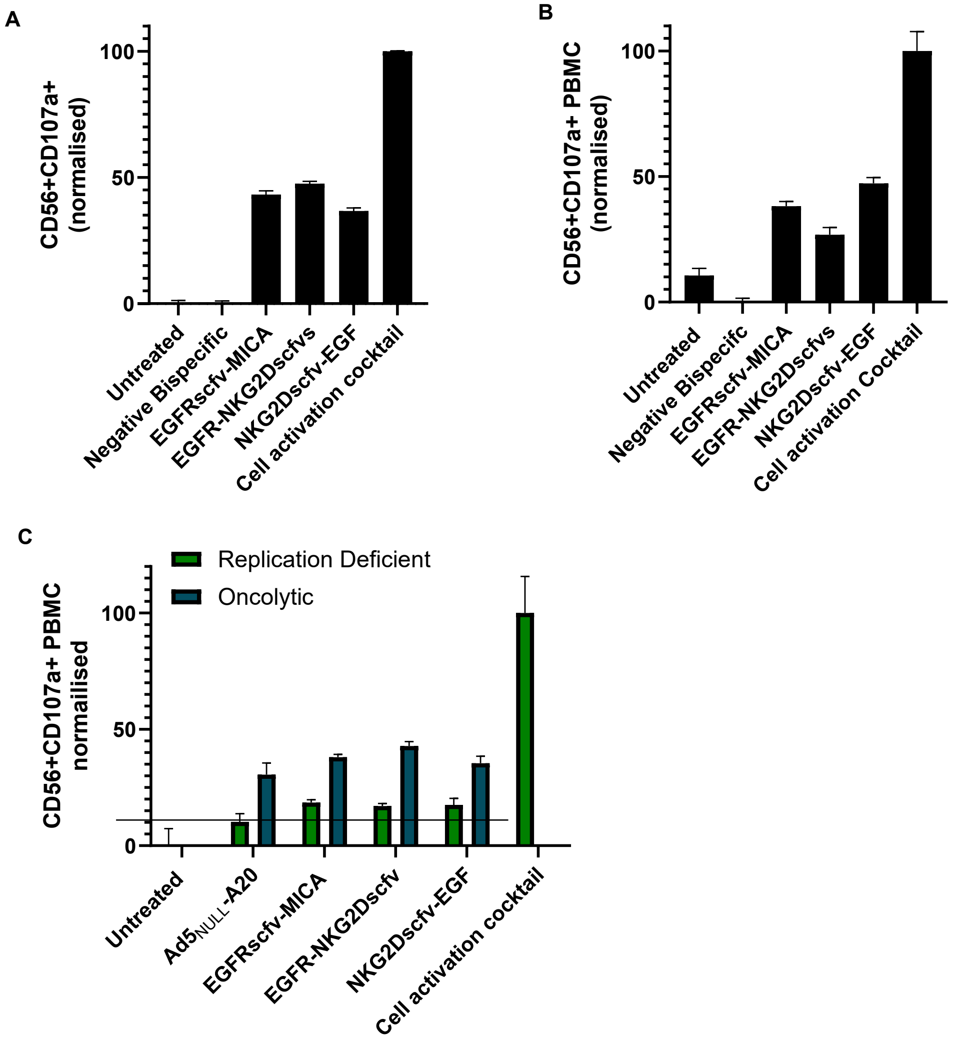

Figure S5. Validation of MICA-EGFRscfv, NKG2Dscfv-EGF and NKG2D-EGFRscfvs *in vitro.* (A) Normalised percentage of CD56+CD107+ cells incubated with MICA-EGFRscfv, NKG2Dscfv-EGF and NKG2D-EGFRscfvs containing supernatants incubated on CHO-EGFR in the presence of NK line (**Fig. 2E**). Data normalised to negative BICA. Triplicate mean and ±SD shown. **(B)**. Percentage of $CD56^+$ $CD107a^+$ NK cells incubated with EGFRscfv-MICA, NKG2Dscfv-EGF and EGFR-NKG2Dscfvs containing supernatants on BT20 cells and co-cultured with PBMC, 6hrs. Data normalised to negative BICA. Triplicate mean and ±SD shown. **(C)**. BT20 cells infected with replication-deficient (RD) and oncolytic (Onc) $Ad5_{NULL}$-A20 EGFRscfv-MICA, NKG2Dscfv-EGF and EGFR-NKG2Dscfvs and co-cultured with isolated NK cells (E:T 1:2). Data normalised to untreated cells. Triplicate mean and ±SD shown.

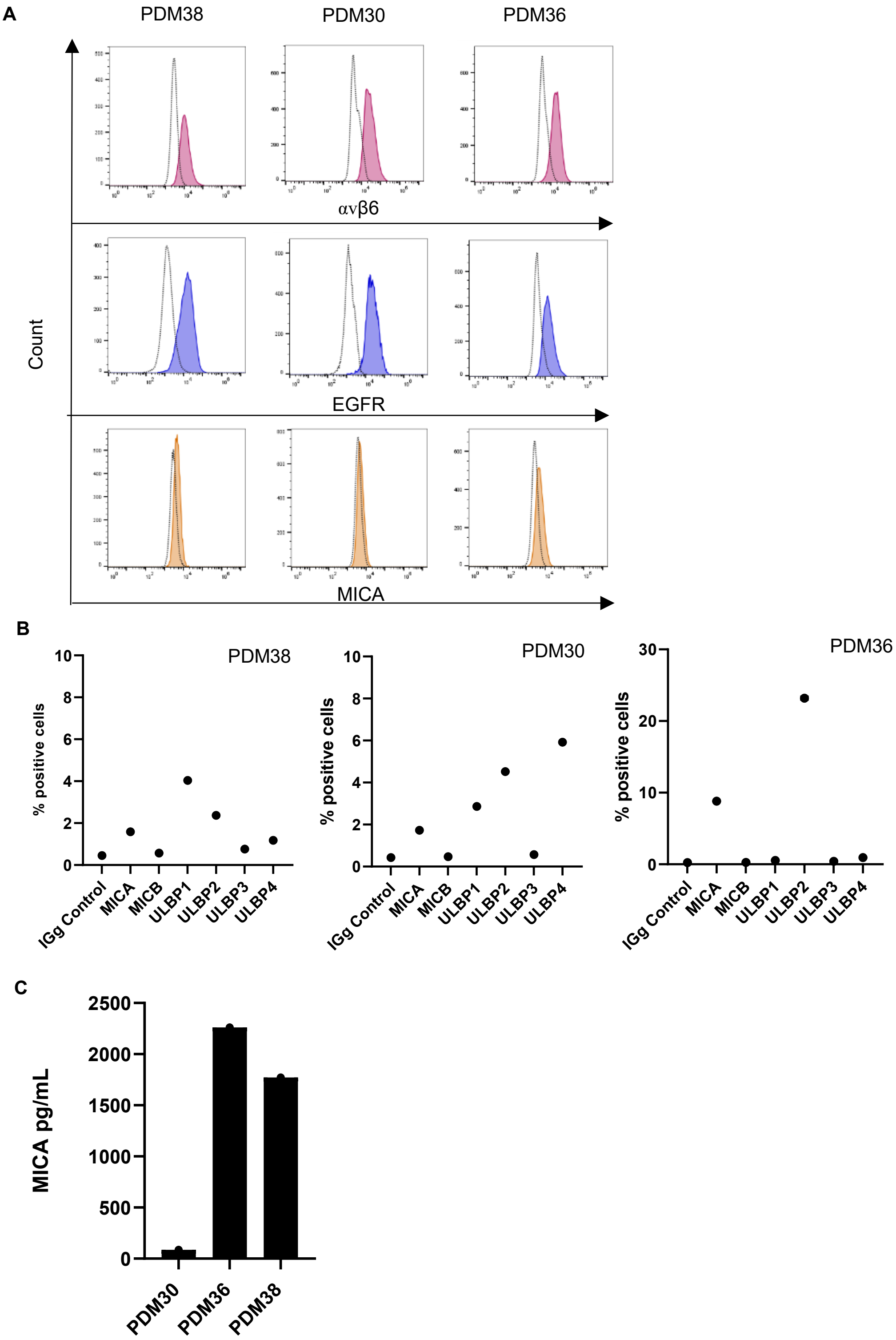

Figure S6. Characterisation of Pancreatic Organoids. (A). Receptor staining of pancreatic organoids PDM38, PDM30 and PDM36 cultured without EGF. Patient derived pancreatic organoids were digested into single cell suspension and stained for surface receptors αvβ6, EGFR and MICA and analysed by flow cytometry. **(B)**. MICB/ULBP1-4 staining of PDM38, PDM30 and PDM36. **(C)** MICA ELISA. Levels of soluble MICA in supernatants of pancreatic organoid in culture. Levels of MICA (pg/mL) interpolated from a standard curve.

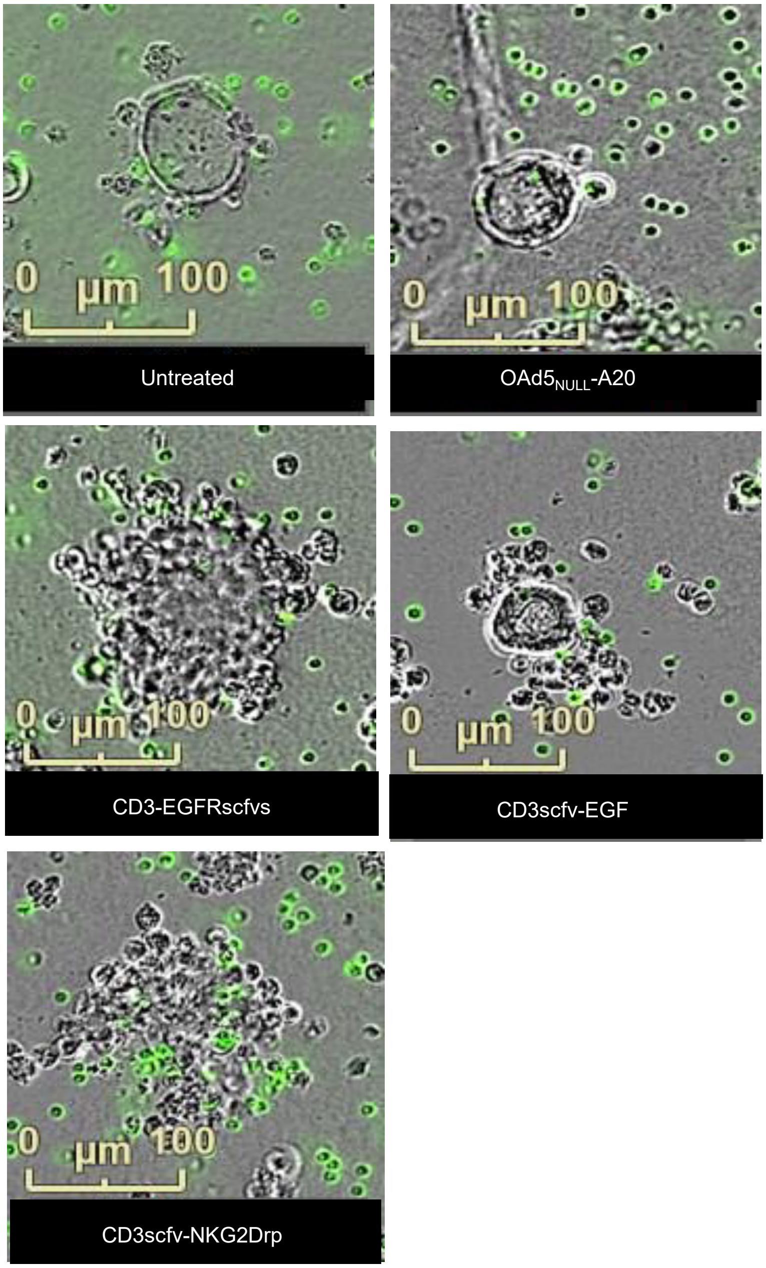

Figure S7. T-cell infiltration of Pancreatic Organoids. T-cells pre-stained with CFSE proliferation dye were co-cultured with PDM38 PDAC organoids (1:5) transduced with $OAd5_{NULL}$-A20.CD3.BICA and monitored in real-time using Incucyte software. 120hr timepoint shown. Scale bar = 100µM.

Table S1. Cell line characteristics and relevance to study

Cell line	Source	Cell type	αvβ6	EGFR	MICA	Relevance to study
293-TRex	Invitrogen	Human Embryonic Kidney	N/A	N/A	N/A	Propagation of Ad5 viruses (CAR+ve)
293-β6	In-house	Human Embryonic Kidney	+++	N/A	N/A	Propagation of $Ad5_{NULL}$-A20 viruses
A431	Arwyn Jones	Epidermoid cancer	N/A	+++	N/A	EGFR +ve cell line for EGFRscfv /EGF binding
BT20	Lynda Coughlan	Triple negative breast cancer	+++	++	+	Solid tumour, permissive to $Ad5_{NULL}$-A20, express TAAs
CHO-K1	ATCC	Chinese hamster ovary	N/A	-	N/A	-ve control line (EGFR-ve) to evaluate specificity/binding of EGFRscfv/EGF BICA
CHO-EGFR	In-house	Chinese hamster ovary	N/A	++	N/A	+ve EGFR cell line to test specificity of EGFRscfv/EGF BICA
HF-CAR	In-house	Human fibroblasts	N/A	N/A	N/A	Permissible to Ad5 (CAR+ve) to produce high concentration BICA S/N
Jurkat	Simon Kollnberger	T-cell	N/A	N/A	N/A	CD3 +ve T-cell reporter to measure BICA function
KYSE30	ATCC	Oesophageal cancer	+++	+++	++	Solid tumour type, permissive to $Ad5_{NULL}$-A20, express TAAs
Panc0403	ATCC	Pancreatic ductal adenocarcinoma	++	++	++	Solid tumour type, permissive to $Ad5_{NULL}$-A20, express TAAs
PT45	ATCC	Pancreatic ductal adenocarcinoma	-	++	+	Non-permissive to $OAd5_{NULL}$-A20,<br>-ve control for oncolytic activity
SKBR3	Arwyn Jones	Breast cancer	N/A	N/A	-	MICA -ve cell line for CD3-NKG2Drp binding
UMSCC4	Ned Powell	Squamous cell carcinoma	++	++	+	Permissive to $OAd5_{NULL}$-A20
U373-MICA	Ceri Fielding	Astrocytoma	N/A	N/A	+	MICA +ve cell line for CD3-NKG2Drp binding

Table S2. Bispecific immune cell activator construct designs

Name	Feature	Identifier/Sequence	Resource
**Adenovirus Type 5**	Homology Right	GGATCCACGTCACTATTGTATACTATATTATACTCTATGTTATACTCTGTAATCCTACTCAATAAACGTGTCACGCC	
**Adenovirus Type 5**	Homology Left	AACCGTCAGATCGCCTGGAGACGCCATCCACGCTGTTTTGACCTCCATAGAAGACACCGGGACCGATCCAGCTGGATCCGCCACC	
**CD33**	Peptide signal	ATGCCGCTGCTACTGCTGCCCCTGCTGTGGGCAGGGGCGCTAGCT	
**$(G_4S_1)_3$ VL/VH**	VL/VH linker A	GGTGGCGGAGGGAGTGGTGGCGGAGGGAGCGGTGGCGGAGGAGT	
**$(G_4S_1)_3$ VH/VL**	VH/VL linker B	GGTGGAGGGGGATCTGGGGGAGGTGGCTCGGCGGTGGTGGTAGT	
**G_4S_1**	Scfv Linker	GGCGGCGGGGGAAGT	
**V5**	Tag	TACCCCTACGACGTGCCCGACTACGCC	
**CD3 (UCHT1)**	Heavy chain	GenBank: AJ853735.1 (Nt:427-792)	NCBI
	Light chain	GenBank: AJ853735.1 (Nt:61- 381)	NCBI
**CD16 (3G8)**	Heavy chain	GenBank: AY173025.1 (Nt:1-354)	NCBI
	Light chain	GenBank: AY173024.1 (Nt:1-332)	NCBI
**EGFR**	Heavy chain	Cetuximab (1-119)	KEGG Drug database
	Light chain	Cetuximab (1-107)	KEGG Drug Database
**NKG2Dscfv**	Heavy chain	Kwong et al 2008	J.Mol.Biol
	Light chain	Kwong et al 2008	J.Mol.Biol
**MICA ligand**	Extracellular (001 allele)	UniPROT: >sp|Q29983|24-307	UniPROT
**EGF ligand**	Extracellular	UniPROT: >sp|P01133|971-1023	UniPROT
**NKG2D receptor**	Extracellular	UniPROT: >sp|P26718|73-216	UniPROT

Table S3. Recombineering Primers

Primer	5’-3’ Sequence
**Homology F**	TCAGATCGCCTGGAGAGACGCC
**Homology R**	CACAGGCGTGACACGTTTATTGAG
**PCR F**	TCAGATCGCCTGGAGACC
**PCR R**	CTAACGCTGCAAGAGTGGGTT
**Sequence F**	CCATGGTGATGCGGTTTT
**Sequence R**	AACTACATAAGACCCCCACC

Table S4. $Ad5_{NULL}$-A20 and Ad5 viral titre

Virus	Bispecific immune cell activator	VP/mL	Pfu/mL	Nanosight (vp/mL)	Particle size (nm)
**$Ad5_{NULL}$-A20 replication deficient**	Anti-CD3scfv-anti-EGFRscfv	9.46E+11	3.5E+09	3.01E+11	96.8
	Anti-CD3scfv-EGF	1.49E+12	3.2E+09	3.65E+11	98.2
	Anti-CD3scfv-NKG2Drp	2.74E+12	4.8E+09	7.90E+11	96.8
	Anti-CD16scfv-anti-EGFRscfv	1.18E+12	3.4E+09	4.31E+11	100.3
	Anti-CD16scfv-EGF	5.21E+11	4.6E+08	9.80E+10	103.1
	Anti-EGFRscfv-MICA	7.38E+11	4.1E+08	1.33E+11	101.9
	EGFrev-MICA	2.59E+12	4.5E+09	1.77E+11	99.8
	Anti-EGFRscfv-anti-NKG2Dscfv	1.60E+12	4.5E+09	3.54E+11	103.3
	Anti-NKG2Dscfv-EGF	3.80E+12	3.5E+09	3.15E+11	102.4
	Anti-CD16scfv-NKG2Drp	4.08E+12	1.1E+09	4.03E+11	102.0
**$Ad5_{NULL}$-A20 Oncolytic**	Anti-CD3scfv-anti-EGFRscfv	3.07E+11	8.91E+08	2.82E+10	98.0
	Anti-CD3scfv-EGF	1.04E+12	2.93E+09	1.60E+11	100.7
	Anti-CD3scfv-NKG2Drp	5.49E+11	1.47E+09	1.07E+11	98.4
	Anti-CD16scfv-anti-EGFRscfv	6.41E+11	1.25E+10	1.34E+11	102.7
	Anti-CD16scfv-EGF	2.65E+11	1.33E+08	2.82E+10	98.0
	Anti-EGFRscfv-MICA	7.98E+11	2.29E+10	1.69E+11	98.0
	EGFrev-MICA	4.89E+11	1.61E+10	1.93E+11	97.8
	Anti-EGFRscfv-anti-NKG2Dscfv	5.89E+11	1.40E+10	9.08E+10	102.2
	Anti-NKG2Dscfv-EGF	1.38E+12	2.77E+10	4.23E+11	101.4
	Anti-CD16scfv-NKG2Drp	1.22E+12	1.86E+10	1.45E+11	97.0
**Ad5 replication deficient**	Anti-CD3scfv-anti-EGFRscfv	7.53E+12	2.26E+10	1.53E+12	102.6
	Anti-CD3scfv-EGF	3.79E+12	1.53E+10	7.09E+11	100.3
	Anti-CD3scfv-NKG2Drp	2.94E+12	1.21E+10	4.34E+11	104.0
	Anti-CD16scfv-anti-EGFRscfv	4.89E+12	1.41E+10	1.05E+12	101.6
	Anti-CD16scfv-EGF	2.14E+12	9.8E+09	3.21E+11	101.7
	Anti-EGFRscfv-MICA	2.74E+12	1.14E+10	4.98E+11	105.8
	EGFrev-MICA	5.26E+12	1.3E+10	9.34E+11	103.7
	Anti-EGFRscfv-anti-NKG2Dscfv	3.44E+12	3.20E+10	8.50E+11	102.6
	Anti-NKG2Dscfv-EGF	2.09E+12	9.4E+09	2.10E+11	102.1
	Anti-CD16scfv-NKG2Drp	3.24E+12	6.1E+09	3.23E+11	102.7

Table S5. Patient Pancreatic Organoid properties

ATCC No.	Tumour Diagnosis	TNM stage	Clinical Stage	Grade*	Therapy	Disease
ATCC No.PDM-38™ HCM-CSHL-0091-C25	Pancreatic Adenocarcinoma Ductal Type	T2N1M0	IIB	G2	Surgery, Chemotherapy Radiotherapy	Progressive
ATCC No. PDM-30™ HCM-CSHL-0089-C25	Pancreatic Adenocarcinoma Ductal Type	T3N2M0	III	G2	Surgery, Chemotherapy	None
ATCC No. PDM-36™ HCM-CSHL-0079-C25	Pancreatic Adenocarcinoma Ductal Type	T2N2M0	III	G3	Surgery, Chemotherapy	Progressive

*Histological
